# No Preference for Performance: Host Plant Preference, Offspring Performance and Host Plant Distribution in the Butterfly *Aricia artaxerxes*

**DOI:** 10.1002/ece3.73998

**Published:** 2026-07-28

**Authors:** Vanda Larsson Åberg, Jesper Boman, Niclas Backström, Martin I. Lind

**Affiliations:** ^1^ Department of Environmental and Biosciences Halmstad University Halmstad Sweden; ^2^ Evolutionary Biology, Department of Ecology and Genetics Uppsala University Uppsala Sweden; ^3^ Animal Ecology, Department of Ecology and Genetics Uppsala University Uppsala Sweden

## Abstract

The connection between female host plant preference and offspring performance is important for understanding how relationships between plants and phytophagous insects have evolved. According to the preference‐performance hypothesis, female insects should evolve to oviposit on host plants on which offspring performance is the highest. Here, we examined the preference‐performance hypothesis in the northern brown argus (*Aricia artaxerxes*) butterfly in the province of Uppland, Sweden, by comparing female host plant preference and larval growth between the host plant species wood cranesbill ( *Geranium sylvaticum* ) and bloody cranesbill ( *G. sanguineum* ). We also investigated if host plant preference in *A. artaxerxes* was related to the geographic distribution of *A. artaxerxes* and its host plants in the province of Uppland. We found that the *A. artaxerxes* females, contrary to the preference‐performance hypothesis, preferred ovipositing on *G. sylvaticum* , even though larvae feeding on *G. sylvaticum* were slightly smaller than those feeding on *G. sanguineum* . Since *G. sylvaticum* is more abundant and probably more utilized than *G. sanguineum* in Uppland, an explanation for this negative preference‐performance connection may be that there are advantages associated with utilizing a more common host plant species, even though larvae feeding on this plant show reduced growth rates. Overall, the results show that factors other than offspring performance, such as geographic distribution, may influence female host plant preference in *A. artaxerxes*.

## Graphical abstract

We tested the preference–performance hypothesis in the butterfly *Aricia artaxerxes* by comparing oviposition preference and larval growth on its two host plants *Geranium sylvaticum* and *Geranium sanguineum* in Uppland, Sweden. Females preferred *G. sylvaticum* despite slightly lower larval growth relative to *G. sanguineum* . This mismatch suggests host plant abundance and geographic distribution may influence oviposition more than offspring performance.

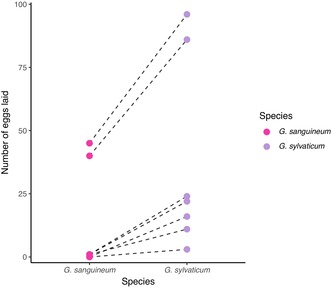

## 1 Introduction

Coevolution is the reciprocal evolutionary change occurring between interacting species through natural selection (Thompson 1989). These interacting species can be mutualists, such as flowering plants and pollinators (Bronstein et al. 2006), or antagonists, such as competitors (Connell 1980), predators and prey (Abrams 2000), and parasites and hosts (Anderson and May 1982). One example of antagonistic coevolution is that of plants and phytophagous (plant‐eating) insects (Ehrlich and Raven 1964). Together, plants and insects compose more than half of the world's described species (Futuyma and Agrawal 2009; IUCN 2025). However, phytophagous insect species generally only utilize a restricted number of host plant species (Wiklund 1975; Bernays and Graham 1988; Thompson 1993). These host plant associations are shaped by plant secondary metabolites involved in the defense against herbivores (Ehrlich and Raven 1964; Bennett and Wallsgrove 1994). According to Ehrlich and Raven's (1964) *escape‐and‐radiate coevolution* hypothesis, plants evolve new secondary metabolites that enable them to escape from herbivores and undergo radiation. When phytophagous insects eventually overcome these defenses and adapt to the plant clade, they sometimes undergo adaptive radiation, resulting in a number of related insect species utilizing related host plant species (Futuyma and Agrawal 2009).

The association between host plant preference and offspring performance is important for understanding how relationships between plants and phytophagous insects have evolved (Thompson 1988; Nylin et al. 1996). In many phytophagous insects, host plants are chosen by adult females during oviposition (Mayhew 1997, Jones 2022). Female host plant preference can be defined as the hierarchal ordering of different host plant species during oviposition and may reflect host plant qualities such as nutritional value and the occurrence of natural enemies (Thompson 1988; West and Cunningham 2002). Such preference is important for offspring performance, influencing their survival, growth, and reproduction. Consequently, the selection of suitable sites for oviposition is crucial for offspring fitness and is the only form of parental care shown by many insects (Renwick 1989; Nylin et al. 1996; Gamberale‐Stille et al. 2014; Jones et al. 2019). According to the *preference‐performance* hypothesis, also known as the *mother knows best* hypothesis (Gripenberg et al. 2010), females should therefore evolve to oviposit on host plants on which offspring performance is the highest (Wiklund 1975; Jaenike 1978).

While two meta‐analyses have demonstrated that female host plant preference generally is correlated to offspring performance, they also highlight that many studies have found poor preference‐performance correlations (Mayhew 1997; Gripenberg et al. 2010). A meta‐analysis separating native and exotic host plant species indicated that the preference‐performance hypothesis was applicable to native host plants, but not necessarily to exotic ones (Jones et al. 2019). However, host plants that are less suitable as food may still be beneficial to utilize if, for example, they reduce exposure to natural enemies (Björkman et al. 1997) or constitute a source of mutualists (Atsatt 1981), providing alternative explanations for poor preference‐performance correlations. In a study on the oviposition of the butterfly *Ogyris amaryllis*, for example, host plants with ant mutualists were preferred over those without ants regardless of plant quality and abundance (Atsatt 1981). Utilizing a more drought resistant host plant species associated with reduced offspring performance can also be beneficial (Näsvall et al. 2021). Moreover, females do not only choose host plants based on the optimization of their offsprings' fitness, but also on the optimization of their own fitness (Nylin et al. 1996; Scheirs et al. 2000). Abundant host plant species associated with an increased oviposition rate may therefore be preferred by females at the cost of individual offspring performance (Nylin et al. 1996; Mayhew 1997). This can be seen as examples of a parent‐offspring conflict (Gamberale‐Stille et al. 2014).

**FIGURE 1:**
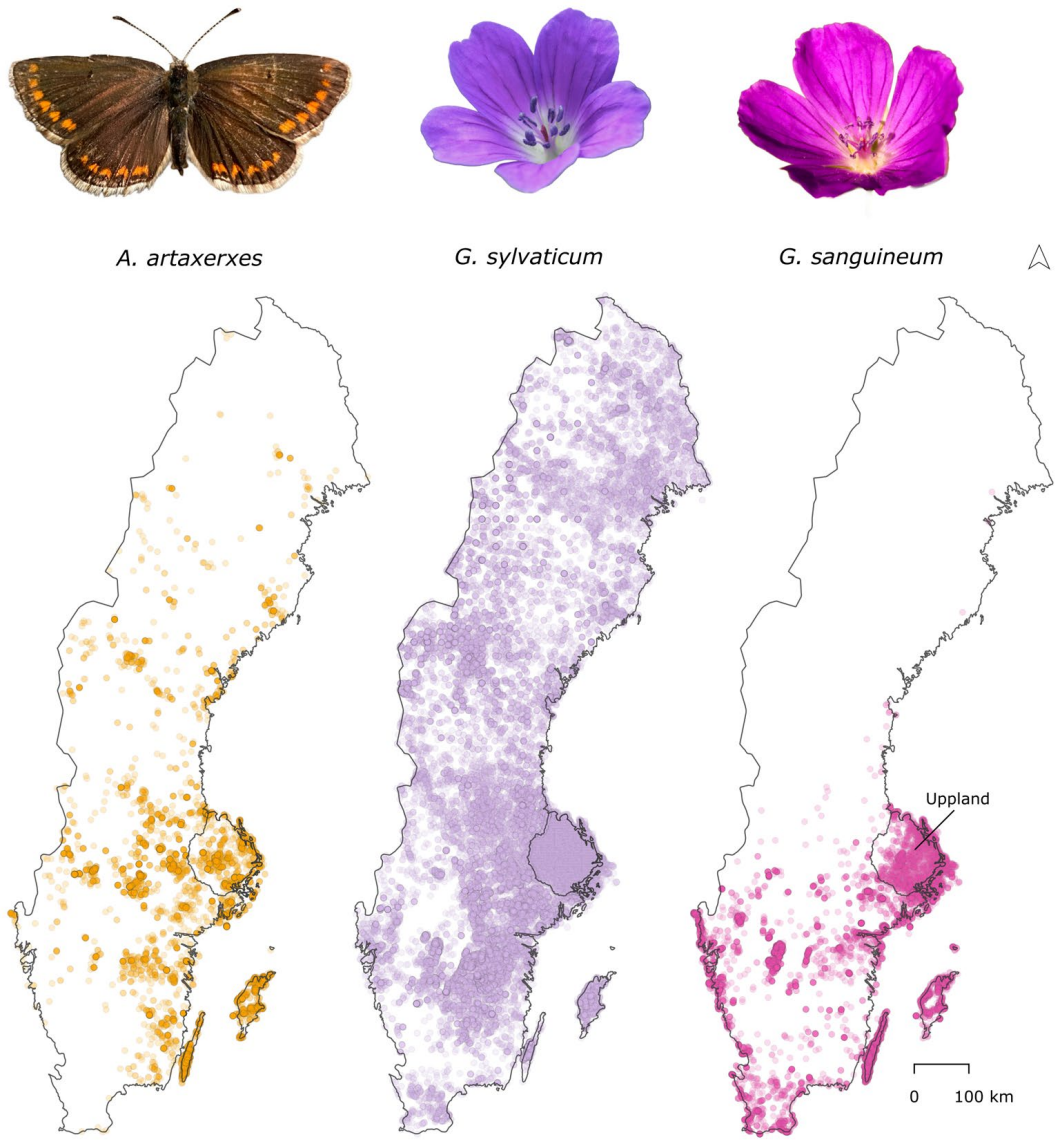
Reported findings of *A. artaxerxes* and the host plants *G. sylvaticum* and *G. sanguineum* in Sweden between year 2000 and 2025, downloaded from the citizen science database *Species Observation System* (*Artportalen*) (SLU Artdatabanken, n.d.).

Many phytophagous insects utilize different host plant species in different parts of their distribution area, but it is unknown if geographic differences in host plant specialization mainly depend on differences in the availability of host plant species or differences in host plant preference (Fox and Morrow 1981, Thompson 1993). The northern brown argus butterfly (*Aricia artaxerxes*) occurs throughout almost all of Sweden, where it utilizes at least two host plant species—wood cranesbill ( *Geranium sylvaticum* ) and bloody cranesbill ( *G. sanguineum* ) (Eliasson et al. 2005) (Figure 1). Common rockrose ( *Helianthemum nummularium* ), which is used by the closely related *A. agestis*, and hoary rockrose (*H. oelandicum*) have also been mentioned as a host plant of *A. artaxerxes* in Sweden (Eliasson et al. 2005). However, rockroses may be utilized exclusively by the southern lineage *horkei*, which was previously described as a subspecies of *A. artaxerxes* on the Baltic Sea island Öland, but is now known to be a hybrid lineage between *A. artaxerxes* and *A. agestis* (Høegh‐Guldberg 1974; Eliasson et al. 2005; Boman et al. 2025). Due to the geographic distribution of its host plant species, it is believed that *A. artaxerxes* utilizes different *Geranium* species in different parts of Sweden, although no studies have yet confirmed this (Eliasson et al. 2005). In the province of Uppland in central Sweden, where this study was conducted, both *G. sylvaticum* and *G. sanguineum* occur, with the former being more frequent than the latter (Eliasson et al. 2005; Jonsell 2010) (Figure 1). The relative usage of the two *Geranium* species by *A. artaxerxes* in Uppland has so far not been investigated in depth.

Here, we use *A. artaxerxes* in the province of Uppland, Sweden to investigate (i) if there is a female host plant preference between the most commonly occurring host plant ( *G. sylvaticum* ) and a rarer host plant ( *G. sanguineum* ). We also test (ii) if there, in accordance with the preference‐performance hypothesis, is a positive connection between female host plant preference and offspring performance. Lastly, we investigate (iii) if host plant preference in *A. artaxerxes* is related to the geographic distribution of *A. artaxerxes* and its host plants *G. sylvaticum* and *G. sanguineum* in Uppland.

## 2 Materials and Methods

### 2.1 Host Plant Preference

For the host plant preference test, *A. artaxerxes* females were collected in the Uppsala area in the province of Uppland, Sweden, between July 2–13, 2024 (localities: 59.8359 N, 17.5880 E; 59.8595 N, 17.5783 E & 59.8394 N, 17.5566 E). The two host plant species *G. sylvaticum* and *G. sanguineum* were also collected in the province of Uppland (localities: 59.8359 N, 17.5880 E; 59.8595 N, 17.5783 E; 59.8394 N, 17.5566 E & 60.5093 N, 17.5973 E). During the field work, we observed that both *G. sylvaticum* and *G. sanguineum* were present in the localities where the butterflies were collected. We placed the collected *A. artaxerxes* females (*n* = 28) individually in 40 × 40 × 40 cm mesh cages with plastic roofs (*n* = 4). However, only a minority of the females oviposited during the host plant preference test, resulting in 7 experimental replicates. We cut the host plants to similar sizes, approximately 30 cm in height, and placed them individually in plastic containers with water. In each cage, we placed four containers with plants, one in each corner with the same host plant species in opposite corners. We switched the placement of the host plant species between the cages. In the centre of each cage, we placed a feeding station consisting of *Cirsium* flowers and sugar water. The host plant preference test ran for 72 h with an 18‐h light +6‐h darkness regime (light between 3 a.m. and 9 p.m. and dark between 9 p.m. and 3 a.m., which approximately corresponds to conditions in Uppland at the time of collection). After the 72 h, we counted the number of eggs laid by each female on each host plant species.

### 2.2 Larval Growth

During the host plant preference test, 346 eggs were laid by the 7 *A. artaxerxes* females. The eggs were laid individually (as opposed to in clusters). The larvae hatching from the eggs were raised on the same host plant as the egg was laid. Host plants were replaced regularly so that larvae always had access to fresh food. If host plants were about to dry up, we placed new plants next to them in the containers. The larvae were raised under the same light conditions used during the host plant preference test (18 h light +6 h dark). Beginning on day 8 after the start of the host plant preference test, we photographed the larvae using a Lumenera Infinity 2–5C microscopy camera mounted on a Leica M165C stereo microscope and the software Infinity analyze version 6.2 (Lumenera corporation 2013). We photographed each cohort of larvae every 3rd day for 12–15 days. For each day and cohort, we photographed up to 20 randomly chosen larvae (5 larvae per plant). To minimize handling, we photographed the larvae on the host plants. We measured the surface area (mm^2^) of the photographed larvae using the polygon selections tool in the software ImageJ version 1.54 (Schneider et al. 2012). In total, we took 370 size measurements.

### 2.3 Geographic Distribution

For the geographic distribution, we downloaded data of reported observations of *A. artaxerxes*, *G. sylvaticum* and *G. sanguineum* in the province of Uppland, Sweden, from the citizen science database *Species Observation System* (*Artportalen*) (SLU Artdatabanken, n.d.) on November 27, 2024. For the distribution of *A. artaxerxes*, we used 1431 observations reported between years 2000 and 2024. For the distribution of *G. sylvaticum* and *G. sanguineum* , we also used data from an inventory of the plants of Uppland (Jonsell 2010). The plant inventory was conducted between years 1991 and 2005 in 2742 grid cells of 2.5 × 2.5 km covering the province of Uppland. The downloaded data showed the grid cells in which *G. sylvaticum* and *G. sanguineum* were noted during the inventory. We created a map of the distribution of *A. artaxerxes*, *G. sylvaticum* and *G. sanguineum* in Uppland using the 2.5 × 2.5 km grid cells from the *plant* inventory in the GIS software QGIS version 3.40 (QGIS Development Team 2024).

### 2.4 Statistical Analyses

The host plant preference, larval growth and geographic distribution were analyzed in the statistical software R version 4.4.1 (R Core Team 2024). Host plant preference was analyzed using a Poisson generalized linear mixed‐effects model (GLM) with the number of eggs laid as the response variable and host plant species ( *G. sylvaticum* and *G. sanguineum* ) as the explanatory variable. The Poisson GLM was chosen because of the count data property of the response variable (the number of eggs laid). Female was used as a random factor to account for the fact that the same female chose between both host plant species, which meant that the samples were not independent. The model was implemented using the *lme4* package, and significances were assessed using the Anova function of the *car* package. The model was evaluated for overdispersion and zero‐inflation using the *DHARMa* package. Larval growth was analyzed with linear mixed‐effects models (LMM) using the *lme4* package (Bates et al. 2015), where size (mm^2^) was the response variable and host plant species ( *G. sylvaticum* and *G. sanguineum* ) and number of days since oviposition (Day in models) were the explanatory variables. Day^2^ was added to the model to account for non‐linear growth. Female (mother) was again used as a random factor since the samples were not independent. The response variable (size, mm^2^) was log transformed since the variance increased with the mean. Since the offspring were kept in groups on the plants where females had laid their eggs, there is a possibility that the density of offspring could affect their growth by competitive interactions. Therefore, in a separate model, we added Density as another factor. Day and Density were scaled to a mean of 0 and a variance of 1, since they were on different scales. Model simplification was performed where non‐significant interactions were removed until Akaike information criterion (AIC) stopped decreasing and the model with lowest AIC was chosen (refitted using maximum likelihood, AIC of all models are presented in Tables S1 and S3). Although some interaction terms were not individually significant, models including these terms sometimes had lower AIC and were therefore retained to optimize overall model fit. Normality of residuals of all models was assessed using quantile‐quantile plots and inspection of the residual distribution. Non‐homogeneity of variances (heteroscedasticity) was investigated using the *performance* package.

To investigate any difference between the observed and expected distribution of *A. artaxerxes* based on the distribution of its host plants, the number of grid cells in which *A. artaxerxes*, *G. sylvaticum* , and *G. sanguineum* occurred was analyzed using Pearson's Chi‐squared test. The grid cells where neither *G. sylvaticum* nor *G. sanguineum* occurred were excluded from the analysis. We also investigated the role of spatial autocorrelation of *A. artaxerxes* records. Therefore, we further modeled the association of *A. artaxerxes* with *G. sylvaticum* and *G. sanguineum* using a logistic regression framework. Since *A. artaxerxes* mainly occurred where either only *G. sylvaticum* or both host plant species occurred, but rarely where only *G. sanguineum* occurred alone, we investigated whether the presence of *A. artaxerxes* differed between grid cells where either both host plants or only *G. sylvaticum* was present. The response variable was the presence or absence of *A. artaxerxes* within the 2.5 × 2.5 km grid cells. As a fixed effect, we included a binary predictor indicating whether both host plant species ( *G. sylvaticum* and *G. sanguineum* ) or only *G. sylvaticum* (the reference category) were present within a grid cell. To account for spatial autocorrelation in the butterfly records, we used a binomial generalized additive model (GAM) implemented in the package *mgcv*. We included a two‐dimensional smooth term of the projected cell‐centre coordinates (in SWEREF99 coordinates) using a thin‐plate regression spline. The model was fitted with a binomial error distribution and logit link function using restricted maximum likelihood (REML) for smoothness selection. Spatial autocorrelation was evaluated using Moran's *I* test. Neighbor relationships among grid cells were defined using an eight‐nearest‐neighbor approach (each cell had 8 neighbors), and row‐standardized spatial weights were applied. We calculated Moran's *I* for (i) butterfly occurrence, (ii) residuals from the non‐spatial logistic regression model, and (iii) residuals from the spatial GAM to assess if the spatial model accounted for any spatial structure in the data.

## 3 Results

### 3.1 Host Plant Preference

**FIGURE 2:**
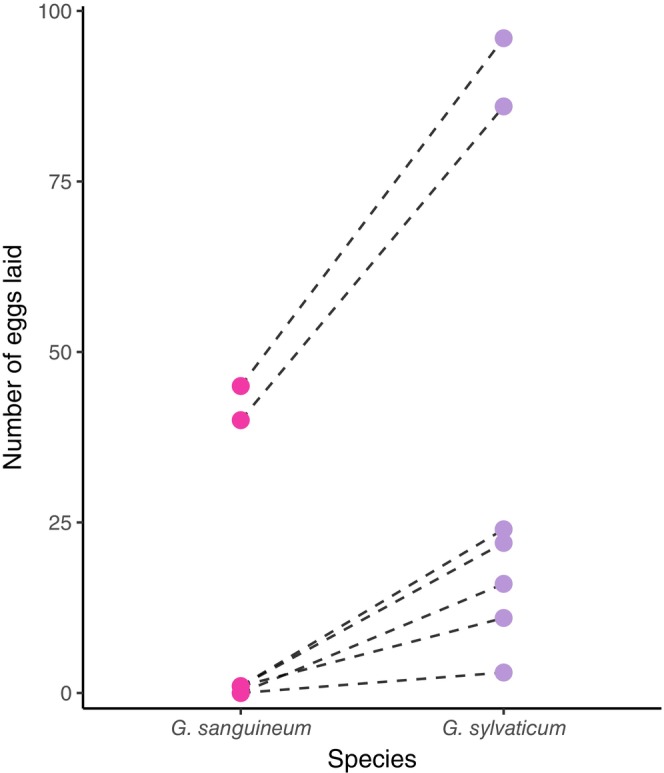
The number of eggs laid by each *A. artaxerxes* female (*n* = 7) on the host plants *G. sanguineum* and *G. sylvaticum* .

We found that the *A. artaxerxes* females laid a significantly higher number of eggs on *G. sylvaticum* compared to *G. sanguineum* (Poisson generalized linear mixed‐effect model: *χ* ^2^ = 76.623, df = 1, *p* < 0.001, Figure 2). Of the seven females that laid eggs, five females oviposited on both *G. sylvaticum* and *G. sanguineum* and two females oviposited exclusively on *G. sylvaticum* . The model had no evidence of overdispersion (dispersion 0.0301, *p* = 0.864) or zero‐inflation (*p* = 0.328).

### 3.2 Larval Growth

**FIGURE 3:**
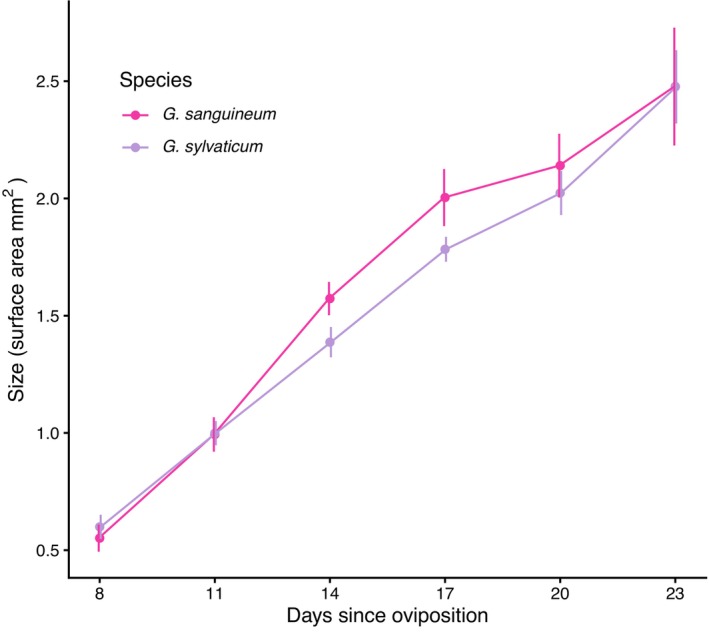
Growth curves of *A. artaxerxes* larvae feeding on either *G. sylvaticum* or *G. sanguineum* on days 8–23 since oviposition. Dots show mean values and bars represent standard errors.

We found a significant effect of host plant species on the growth of the *A. artaxerxes* larvae, demonstrating that larvae feeding on *G. sylvaticum* were smaller than larvae feeding on *G. sanguineum* (Table 1; Table S2; Figure 3). There was, however, no interaction between the incline or curvature of the growth between the larvae feeding on *G. sylvaticum* and the larvae feeding on *G. sanguineum* (Table S1). Moreover, we found that the growth of the larvae was non‐linear (decreasing) as indicated by the significant effect of Day^2^ (Table 1; Figure 3). We found no evidence of heteroscedasticity (*p* = 0.311).

**TABLE 1:** *χ* ^2^, df and *p*‐value of the larval growth linear mixed‐effects model with the lowest AIC.

	*χ* ^2^	df	*p*
Intercept	35.87	1	< 0.001
Day	858.44	1	< 0.001
Species	6.42	1	0.011
Day^2^	53.04	1	< 0.001

To investigate the possibility that any growth differences were driven by competition, we fitted a separate set of models that also included the density of larvae. After model simplification (Table S3), we found qualitatively similar results, where larval size was smaller for offspring feeding on *G. sylvaticum* . Larval density did not have any direct effect on size, nor was it included in any significant interactions, although model selection indicated that its inclusion in the final model improved model fit (Table 2; Table S4). Again, we found no evidence of heteroscedasticity (*p* = 0.259).

**TABLE 2:** *χ* ^2^, df and *p*‐values of the larval growth linear mixed‐effects model with the lowest AIC, including the effect of larval density.

	*χ* ^2^	df	*p*
Intercept	29.05	1	< 0.001
Day	266.72	1	< 0.001
Species	4.03	1	0.044
Density	2.65	1	0.103
Day^2^	29.61	1	< 0.001
Day × species	0.60	1	0.441
Day × density	0.01	1	0.929
Species × density	2.25	1	0.134
Species × day^2^	2.41	1	0.121
Density × day^2^	1.99	1	0.159
Day × species × density	2.87	1	0.090

### 3.3 Geographic Distribution

**FIGURE 4:**
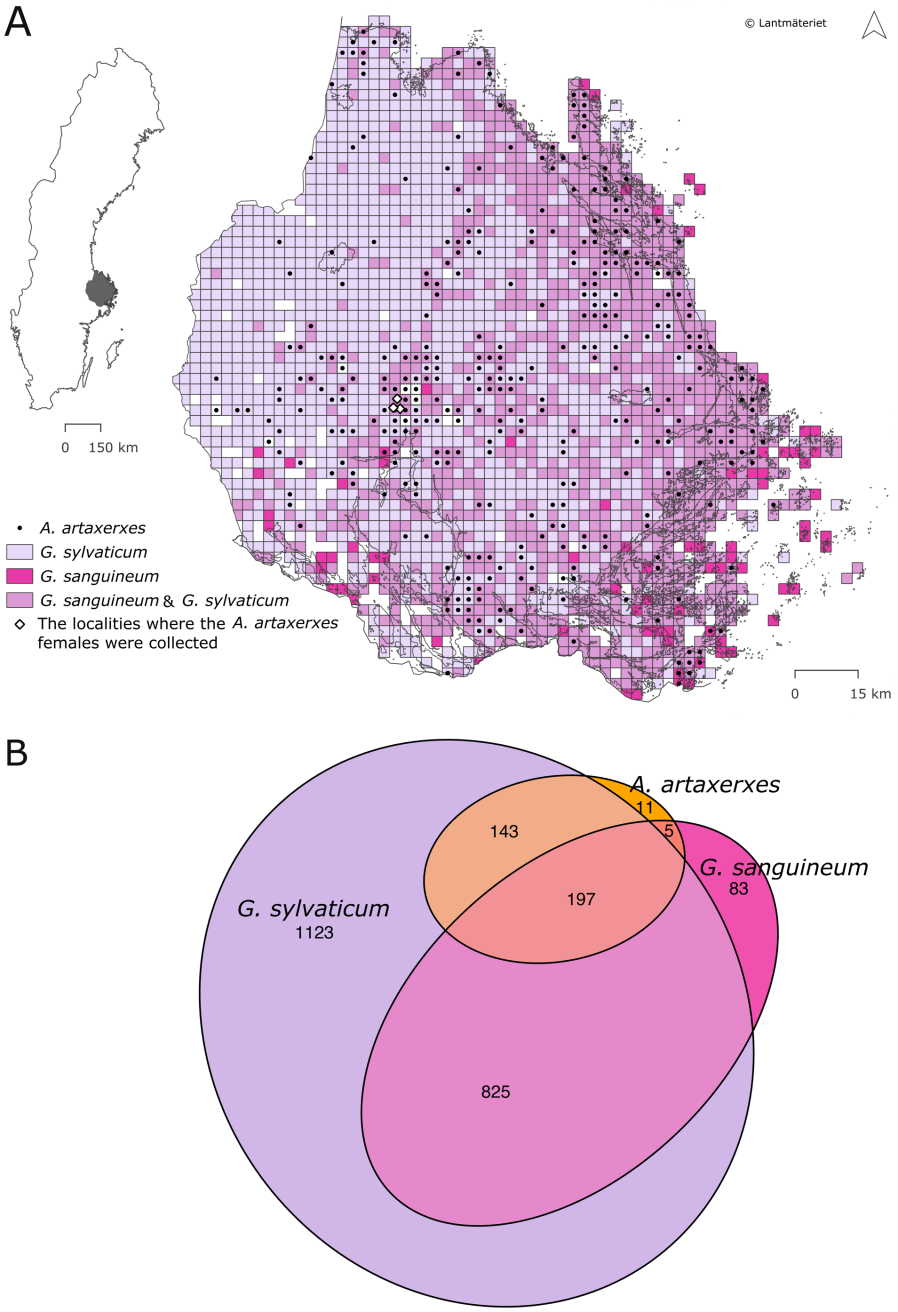
(A) The distribution of *A. artaxerxes*, *G. sylvaticum* and *G. sanguineum* in the province of Uppland, Sweden, in 2.5 × 2.5 km grid cells as well as the localities where the *A. artaxerxes* females were collected for the host plant preference test. The map was generated using a basemap from Lantmäteriet. (B) Venn diagram of the number of grid cells in which *A. artaxerxes*, *G. sylvaticum* and *G. sanguineum* occurred and their overlap.

**FIGURE 5:**
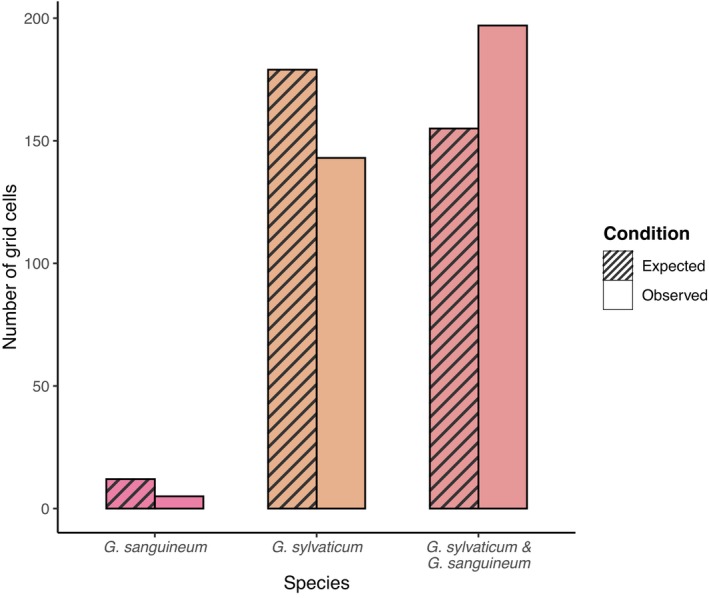
Expected and observed number of 2.5 × 2.5 km grid cells in the province of Uppland, Sweden, where *A. artaxerxes* occurred and where either only *G. sanguineum* , only *G. sylvaticum* or both *G. sylvaticum* and *G. sanguineum* were present.

*G. sylvaticum* and *G. sanguineum* occurred in 96% and 47% of the 2387 2.5 × 2.5 km grid cells, respectively (Figure 4). *A. artaxerxes* commonly occurred where only *G. sylvaticum* was present (in 40% of the grid cells where it was present), but rarely where only *G. sanguineum* was present (in 1% of the grid cells). However, we found that there was a significant difference between the observed and expected distribution of *A. artaxerxes* based on the distribution of *G. sylvaticum* and *G. sanguineum* (Pearson's Chi‐squared test: *χ* ^2^ = 25.973, df = 2, *p* < 0.001, Figure 5). *A. artaxerxes* mainly (in 55% of the grid cells where it was present) occurred in grid cells where both *G. sylvaticum* and *G. sanguineum* were present, and more frequently so than expected (Figures 4 and 5). However, *A. artaxerxes* occurred less frequently than expected where only *G. sylvaticum* or only *G. sanguineum* was present (Figure 5).

We further did an explorative analysis to investigate the role of spatial autocorrelation of the butterfly records, as they (in contrast to the host plant observations) originate from citizen science data. *A. artaxerxes* occurrence exhibited significant positive spatial autocorrelation (Moran's *I* = 0.154, *p* < 0.001). A non‐spatial logistic regression confirmed that *A. artaxerxes* occurrence was higher in grid cells where both host plant species were present than in cells containing only *G. sylvaticum* (*β* = 0.619 ± 0.118 SE, *z* = 5.24, *p* < 0.001).

After accounting for spatial structure using a generalized additive model, the effect of host plant co‐occurrence remained significant but was reduced in magnitude (*β* = 0.326 ± 0.146 SE, *z* = 2.23, *p* = 0.026). The spatial smooth term was highly significant (edf = 35.6, *χ* ^2^ = 182.9, *p* < 0.001), which confirmed the previous finding of substantial spatial structure in the distribution of butterfly records. Inclusion of the spatial smooth reduced residual spatial autocorrelation from Moran's *I* = 0.140 to 0.024, which indicated that most of the spatial structure was accounted for by the latter model, although we still found a weak residual autocorrelation (Moran's *I* = 0.024, *p* = 0.008).

## 4 Discussion

### 4.1 Preference and Performance

Contrary to the preference‐performance hypothesis (Wiklund 1975; Jaenike 1978), we found that *A. artaxerxes* females from the Uppsala area in the province of Uppland, Sweden, strongly prefer ovipositing on *G. sylvaticum* rather than on *G. sanguineum* , even though larvae feeding on *G. sylvaticum* grew slightly smaller than those feeding on *G. sanguineum* . This result held true even when accounting for the higher larval density and possible stronger competitive interactions on *G. sylvaticum* . Our results indicate that there are other factors than offspring performance influencing the *A. artaxerxes* females' host plant preference. Even though female host plant preference generally is positively associated with offspring performance (Mayhew 1997; Gripenberg et al. 2010; Jones et al. 2019; Zanco et al. 2025), many exceptions to the preference‐performance hypothesis have been documented (Wiklund 1975; Chew 1977; Courtney 1981; Valladares and Lawton 1991; Ohsaki and Sato 1994; Underwood 1994; Björkman et al. 1997; Berdegué et al. 1998; Clark et al. 2011; Davis and Cipollini 2014; Näsvall et al. 2021), including this study. Indeed, it can be adaptive for females not to choose the host plant with the highest quality as food for their offspring (Gripenberg et al. 2010). In the green‐veined white ( *Pieris napi* ) (Ohsaki and Sato 1994) and the redheaded pine sawfly ( *Neodiprion sertifer* ) (Björkman et al. 1997), for example, oviposition on less nutritious host plants may reduce exposure to parasites. In the amaryllis azure (*Ogyris amaryllis*), oviposition on a less nutritious host plant species can provide a source of ant mutualists (Atsatt 1981). In the common wood white (*Leptidea sinapis*), females may prefer ovipositing on a less nutritious host plant species due to it being more drought resistant (Näsvall et al. 2021). Sometimes, however, female host plant preference does not seem to be adaptive, for example when females oviposit on exotic host plant species with low offspring survival, such as documented in the old world swallowtail ( *Papilio machaon* ) (Wiklund 1975), the green‐veined white ( *Pieris napi* ) (Chew 1977), and the West Virginia white ( *Pieris virginiensis* ) (Davis and Cipollini 2014). In this way, exotic plants can negatively impact native butterfly species (Graves and Shapiro 2003), and an explanation for this behavior is that adaptations have not yet evolved for females to avoid ovipositing on exotic species (Wiklund 1975; Chew 1977).

Another factor influencing female host plant preference is differences in abundance between host plant species (Rausher 1980; Singer 1983; Nylin et al. 1996; Mayhew 1997; West and Cunningham 2002). When the locally most abundant host plant offers the best offspring performance, this plant should be preferred. This has for example recently been demonstrated in *A. artaxerxes*' close relative the brown argus (*A. agestis*) in the UK, where recently established local populations of this expanding species (Thomas et al. 2001) have switched their oviposition preference from the perennial rockrose ( *Helianthemum nummularium* ) to the more widespread *Geranium molle* , where offspring performance is higher (Zanco et al. 2025). However, female butterflies are also more likely to oviposit on lower quality host plant species if they are more abundant than higher quality host plant species (Rausher 1980, Singer 1983, Nylin et al. 1996, Mayhew 1997, West and Cunningham 2002). Females that search preferentially for a more common host plant species may discover more plants and lay more eggs during their lifetimes compared to females that search preferentially for a rarer host plant species, thereby increasing their fitness (Rausher 1980; Nylin et al. 1996). Abundant host plant species may therefore, even at the cost of individual offspring performance, be preferred by females and trade‐offs may be made between quality and quantity of offspring (Nylin et al. 1996; Mayhew 1997). This may be an important factor shaping the *A. artaxerxes* females' preference for *G. sylvaticum* since *G. sylvaticum* is more commonly occurring than *G. sanguineum* in Uppland, both overall and in areas where *A. artaxerxes* occurs. Further, larvae feeding on *G. sylvaticum* were smaller than larvae feeding on *G. sanguineum* , but only slightly. The potential difference in female fitness based on host plant abundance (Rausher 1980, Nylin et al. 1996) and relatively low difference in offspring performance may explain why the *A. artaxerxes* females, contrary to the preference‐performance hypothesis (Wiklund 1975; Jaenike 1978), do not prefer to oviposit on the host plant species on which offspring performance is the highest. Negative preference‐performance associations, such as the one found in *A. artaxerxes* in our study, may therefore indicate a conflict between female fitness and direct offspring fitness, i.e., a parent‐offspring conflict, where females maximize their fitness by ovipositing on suboptimal host plants at the expense of offspring performance (Gamberale‐Stille et al. 2014). However, while not measured in our study, factors other than host plant abundances can contribute to host plant selection and may result in negative preference‐performance connections, for example plant secondary metabolite and nutritional composition. Regarding secondary metabolites, species in the genus *Geranium*, including *G. sylvaticum* and *G. sanguineum* , produce polyphenols such as tannins, phenolic acids and flavonoids (Tuominen et al. 2013; Ilić et al. 2026). Polyphenols have been identified as oviposition stimulants in several phytophagous insects (Singh et al. 2021). It is possible that *G. sylvaticum* produces stronger chemical cues compared to *G. sanguineum* , stimulating oviposition, while containing lower macronutrient levels such as nitrogen, constraining larval growth. Such cues that stimulate oviposition while impairing offspring performance could constitute a sensory trap for butterfly females (Jones and Agrawal 2019) and could be a fruitful venue for future investigations.

### 4.2 The Role of Host Plant Distribution

When investigating the geographical distribution of *A. artaxerxes* and its host plants, we found that *A. artaxerxes* mainly occurs in areas where both *G. sylvaticum* and *G. sanguineum*, or only *G. sylvaticum* occur, and significantly more frequently where both host plants occur than expected based on the distribution of the two host plants. Moreover, *A. artaxerxes* commonly occurs in areas where only *G. sylvaticum* occurs and, although rarely, where only *G. sanguineum* occurs. It should, however, be noted that the availability of host plants is likely not the only factor determining the realized niche of *A. artaxerxes*, and its common occurrence in areas where both host plant species occur may reflect suitable microclimate and abiotic conditions. Although these results are based on a relatively high number of citizen‐reported *A. artaxerxes* observations (*n* = 1431), the observations are patchily distributed, and the actual distribution of *A. artaxerxes* probably exceeds the distribution as reported in the database, which may affect the results. We did find spatial autocorrelation of the *A. artaxerxes* records, but the signal of spatial autocorrelation was substantially reduced when we used a spatially explicit model, which still confirmed a significant positive effect of host‐plant co‐occurrence on *A. artaxerxes* presence. In contrast to the butterfly records, the distributions of host plants is based on atlas inventories (Jonsell 2010) and should be considered robust. Still, there are areas where only one host plant species occurs, indicating that there are areas where either *G. sanguineum* or *G. sylvaticum* cannot be used. Regarding areas where both host plant species occur, the host plant preference test indicates that *A. artaxerxes* mainly utilizes *G. sylvaticum* in these areas, but that *G. sanguineum* probably is utilized to a smaller degree as well. In partial agreement with the previous estimation (Eliasson et al. 2005), these results indicate that *A. artaxerxes* mainly, but not exclusively, utilizes *G. sylvaticum* in Uppland. The results also indicate that the *A. artaxerxes* females prefer the host plant species that is most commonly occurring in Uppland. Studies of phytophagous insects using different host plant species in different parts of their distribution area show that some populations have diverged in host plant preference (Gotthard et al. 2004; Näsvall et al. 2021) while others have not (Jaenike 1989; Thompson 1993). The *A. artaxerxes* females' preference for *G. sylvaticum* may therefore be a local adaptation in host plant preference, although studying *A. artaxerxes'* host plant preference in other geographical areas where other host plant species are utilized would be necessary to determine this.

## 5 Conclusions

Contrary to the preference‐performance hypothesis (Wiklund 1975; Jaenike 1978), we found that the *A. artaxerxes* females from the province of Uppland, Sweden, prefer ovipositing on *G. sylvaticum* compared to *G. sanguineum* , even though larvae feeding on *G. sylvaticum* are slightly smaller than those feeding on *G. sanguineum* . Since *G. sylvaticum* is more commonly occurring than *G. sanguineum* in Uppland, potential differences in female fitness based on host plant abundance (Rausher 1980; Nylin et al. 1996) and the low difference in offspring performance may explain why the *A. artaxerxes* females do not prefer to oviposit on the host plant species on which offspring performance is the highest. *Geranium sylvaticum* being the most commonly occurring and probably most utilized host plant in Uppland indicates that the *A. artaxerxes* females' preference for *G. sylvaticum* could be a local adaptation in host plant preference, although further studies are needed to determine this. Overall, the results show that factors other than offspring performance, such as geographic distribution and local abundance of host plant species, may influence female host plant preference in *A. artaxerxes*.

## Author Contributions

**Vanda Larsson Åberg:** conceptualization (supporting), data curation (lead), formal analysis (supporting), investigation (lead), methodology (supporting), visualization (equal), writing – original draft (lead), writing – review and editing (equal). **Jesper Boman:** visualization (supporting), writing – review and editing (equal). **Niclas Backström:** conceptualization (equal), funding acquisition (equal), methodology (lead), resources (equal), supervision (supporting), writing – review and editing (equal). **Martin I. Lind:** conceptualization (equal), formal analysis (lead), funding acquisition (equal), resources (equal), supervision (lead), visualization (equal), writing – original draft (supporting), writing – review and editing (equal).

## Funding

This work was supported by Vetenskapsrådet, 2019‐04791, 2020‐04388, 2025‐00450, Lennanders Foundation, and Birgitta Sintring Foundation.

## Conflicts of Interest

The authors declare no conflicts of interest.

## Supporting information


**Table S1:** Model structure and Akaike information criterion (AIC) of the larval growth linear mixed‐effects models.
**Table S2:** Parameter estimates (mean SE) of the fixed effects in the model with lowest AIC (Day + Day^2^ + Species).
**Table S3:** Model structure and Akaike information criterion (AIC) of the larval growth linear mixed‐effects models including larval density.
**Table S4:** Parameter estimates (mean SE) of the fixed effects in the model with lowest AIC that includes Density.

## Data Availability

Data and code is available at Dryad https://datadryad.org/dataset/doi:10.5061/dryad.rxwdbrvrv.
