# Supplementary material for: No Preference for Performance: Host Plant Preference, Offspring Performance and Host Plant Distribution in the Butterfly Aricia artaxerxes
Source: Ecol Evol. 2026 Jul 28;16(8):e73998. doi: 10.1002/ece3.73998 (PMC13415991; doi:10.1002/ece3.73998)
Supplement: Supplementary file 1 — Table S1: Model structure and Akaike information criterion (AIC) of the larval growth linear mixed‐effects models. Table S2: Parameter estimates (mean SE) of the fixed effects in the model with lowest AIC (Day + Day2 + Species). Table S3: Model structure and Akaike information criterion (AIC) of the larval growth linear mixed‐effects models including larval density. Table S4: Parameter estimates (mean SE) of the fixed effects in the model with lowest AIC that includes Density. [file ECE3-16-e73998-s001.docx]

# Supplementary material

**No preference for performance:**

**Host plant preference, offspring performance and host plant distribution in the butterfly *Aricia artaxerxes***

**Supplementary table 1.** Model structure and Akaike information criterion (AIC) of the larval growth linear mixed-effects models.

| *Model structure* | *AIC* |
| --- | --- |
| Day + Day^2^ + Species + Species:Day + Species:Day^2^ | 269.44 |
| Day + Day^2^ + Species + Species:Day | 269.24 |
| Day + Day^2^ + Species + Species:Day^2^ | 267.81 |
| Day + Day^2^ + Species | 267.45 |

**Supplementary table 2.** Parameter estimates (mean SE) of the fixed effects in the model with lowest AIC (Day + Day^2^ + Species).

| **Parameter** | **Mean** | **SE** |
| --- | --- | --- |
| Intercept | 0.550 | 0.092 |
| Day | 0.521 | 0.018 |
| Species | -0.108 | 0.043 |
| Day^2^ | -0.138 | 0.019 |

**Supplementary table 3.** Model structure and Akaike information criterion (AIC) of the larval growth linear mixed-effects models including larval density.

| *Model structure* | *AIC* |
| --- | --- |
| Day + Species + Density + Day^2^ + Day:Species + Day:Density + Species:Density + Species:Day^2^ + Density:Day^2^ + Day:Species:Density + Day^2^:Species:Density | 257.13 |
| Day + Species + Density + Day^2^ + Day:Species + Day:Density + Species:Density + Species:Day^2^ + Density:Day^2^ + Day^2^:Species:Density | 258.06 |
| Day + Species + Density + Day^2^ + Day:Species + Day:Density + Species:Density + Species:Day^2^ + Density:Day^2^ + Day:Species:Density | 255.15 |
| Day + Species + Density + Day^2^ + Day:Species + Day:Density + Species:Density + Species:Day^2^ + Density:Day^2^ | 256.09 |
| Day + Species + Density + Day^2^ + Day:Species + Species:Density + Species:Day^2^ + Density:Day^2^ | 262.92 |
| Day + Species + Density + Day^2^ + Day:Species + Day:Density + Species:Density + Species:Day^2^ | 256.15 |
| Day + Species + Density + Day^2^ + Day:Density + Species:Density + Species:Day^2^ + Density:Day^2^ | 255.44 |
| Day + Species + Density + Day^2^ + Day:Species + Day:Density + Species:Density + Density:Day^2^ | 256.42 |
| Day + Species + Density + Day^2^ + Day:Species + Day:Density + Species:Day^2^ + Density:Day^2^ | 256.66 |

**Supplementary table 4.** Parameter estimates (mean SE) of the fixed effects in the model with lowest AIC that includes Density.

| **Parameter** | **Mean** | **SE** |
| --- | --- | --- |
| Intercept | 0.556 | 0.103 |
| Day | 0.537 | 0.033 |
| Species | -0.152 | 0.076 |
| Density | 0.070 | 0.043 |
| Day^2^ | -0.185 | 0.034 |
| Day : Species | -0.030 | 0.039 |
| Day : Density | -0.003 | 0.038 |
| Species : Density | -0.078 | 0.052 |
| Species : Day^2^ | 0.063 | 0.041 |
| Density : Day^2^ | -0.028 | 0.020 |
| Day : Species : Density | 0.073 | 0.043 |
